# P-1890. Implementation and outcomes of a fully remote tele-ID and tele-OPAT program at a regional medical center

**DOI:** 10.1093/ofid/ofae631.2051

**Published:** 2025-01-29

**Authors:** Matthew R Davis, Neel Shah, Stephen M Maurer, Megan Gray Neils, Nazia Khan

**Affiliations:** Infectious Diseases Connect ; UPMC, Austin, Texas; University of Pittsburgh Medical Center, Pittsburgh, PA; University of Pittsburgh Medical Center, Pittsburgh, PA; University of Pittsburgh Medical Center, Pittsburgh, PA; University of Pittsburgh Medical Center, Pittsburgh, PA

## Abstract

**Background:**

Infectious diseases (ID) consult services and outpatient parenteral antimicrobial therapy (OPAT) programs have traditionally been restricted to well-equipped academic medical centers. However, the integration of telehealth technologies into ID specialty care now allows for the expansion of ID expertise to underserved medical communities, including non-academic medical centers, and community and rural hospitals. Our initiative pioneered a comprehensive tele-ID service, encompassing both inpatient consultations and a tele-OPAT service for post-discharge management. Herein, we describe the program structure and outcomes from the first year of our integrated tele-ID and tele-OPAT program, implemented at a 144-bed community hospital in Vermont.

**Methods:**

The program featured an inpatient tele-ID consult service supported by local nurse tele-presenters who would facilitate physical exams for a remote ID physician using videoconferencing technology. Patients deemed appropriate for OPAT were referred to the remote tele-OPAT team which consisted of a nurse coordinator, an ID pharmacist, and the current ID physician attending the inpatient service. The nurse coordinator's responsibilities included communicating with the local case management team, home health, and post-discharge facilities to ensure orders were placed, and communications were triaged for the tele-OPAT team. The ID pharmacist reviewed OPAT regimens for appropriateness and monitored outpatient labs for safety and dose adjustments. Tele-OPAT patients were seen locally by the ID physician in a pre-existing clinic with the assistance of a nurse tele-presenter.

**Results:**

Ninety patients were enrolled in the tele-OPAT program in the first twelve months. Of these, 62 (68.9%) completed therapy without adverse event (AE) or 30-day readmission. 30-day readmission occurred in 14 patients (15.6%) and 20 patients (22.2%) had a documented AE during therapy, with 10 patients (11.1%) experiencing line-related issues.

**Conclusion:**

Our telehealth initiative successfully expanded ID expertise to an underserved community hospital, with a majority of patients completing therapy without AEs or readmissions, demonstrating the efficacy and safety of remote ID consultations and tele-OPAT services.

**Disclosures:**

All Authors: No reported disclosures

